# SOX2 Promotes Cell Proliferation and Metastasis in Triple Negative Breast Cancer

**DOI:** 10.3389/fphar.2018.00942

**Published:** 2018-08-21

**Authors:** Peng Liu, Hailin Tang, Cailu Song, Jin Wang, Bo Chen, Xiaojia Huang, Xiaoqing Pei, Longzhong Liu

**Affiliations:** ^1^State Key Laboratory of Oncology in South China, Collaborative Innovation Center of Cancer Medicine, Department of Breast Oncology, Sun Yat-sen University Cancer Center, Guangzhou, China; ^2^State Key Laboratory of Oncology in South China, Collaborative Innovation Center of Cancer Medicine, Department of Ultrasond, Sun Yat-sen University Cancer Center, Guangzhou, China

**Keywords:** SOX2, triple negative breast cancer, proliferation, metastasis, potential target

## Abstract

This study explored the expression, biological function and prognostic role of SOX2 in triple negative breast cancer (TNBC). Quantitative real-time PCR and immunohistochemistry were used to detect the expression of SOX2 in TNBC cell lines and clinical tissues. MTT assay, Transwell assay, flow cytometry and xenograft mouse model were used to assess the biological functions of SOX2. It was found that SOX2 was up-regulated in both TNBC cell lines and clinical tissues. High expression of SOX2 was associated with shorter overall survival and disease free survival. Moreover, inhibition of SOX2 suppressed cell proliferation and invasion, induced cell apoptosis *in vitro*, and suppressed tumorigenesis and metastasis *in vivo*. In addition, analysis of TNM stage and lymph nodes infiltration among the 237 TNBC patients by paired χ^2^ test showed that SOX2 was inversely correlated with tumor status, our findings provided evidence that SOX2 acts as a tumor promoter in TNBC and inhibition of SOX2 could be a potential therapeutic strategy for TNBC.

## Introduction

Triple negative breast cancer (TNBC) is defined by the lack of estrogen receptor (ER), progesterone receptor (PR) as well as the gene expression of human epidermal growth factor receptor 2 (HER2) (Denkert et al., 2016). TNBC accounts for approximately 20% of all breast cancers and is an aggressive breast cancer subtype with poor prognosis (Liedtke et al., 2010). Currently, the main strategy of therapy for TNBC is chemotherapy. However, drug resistance and tumor metastasis occurs frequently (Zhang et al., 2016). Although trastuzumab is an effective targeted therapeutic drug for HER2+ breast cancer, there is no effective specific targeted agent approved for the treatment of TNBC (Yao et al., 2017). Therefore, new targeted strategies for TNBC are urgently needed.

Triple negative breast cancer is a heterogeneous disease characterized by aberrations at genomic or molecular levels resulting in a great multitude of dysregulated signaling pathways (Jiang et al., 2014; Hon et al., 2016). SRY-related HMG box-containing transcription factor-2 (SOX2) is one of these abnormal expressed genes in many cancers including breast cancer (Zheng et al., 2015). SOX2 is a key transcription factor and plays an extremely important role in maintaining pluripotency of stem cells. SOX2 is highly expressed in embryonic tissues while rarely expressed in adult normal somatic cells (Feng and Wen, 2015). As an important cancer stem cell marker, SOX2 is involved in cell proliferation, differentiation, invasion, metastasis, drug resistance, relapse, and others processes of tumors (Saigusa et al., 2009; Yang S. et al., 2015). Such as SOX2 can promote tumor tumorigenesis and development in tongue squamous cell carcinoma, osteosarcoma, or gastric cancer through various signaling pathways (Liu et al., 2018; Luo et al., 2018; Maurizi et al., 2018). Studies show that SOX2 is frequently abnormally expressed in a variety of malignant tumors. For example, SOX2 is not expressed in normal breast tissues but highly expressed in breast cancer tissues (Abd El-Maqsoud and Abd El-Rehim, 2014); SOX2 is highly expressed in normal gastric mucosa, but there is almost no expression of SOX2 in intestinal metaplasia of gastric mucosa (Carrasco-Garcia et al., 2016), suggesting that the expression of SOX2 is tumor specific.

A number of studies have shown that the expression of SOX2 is associated with the prognosis of metastatic or recurrence tumors. The DNA amplification and protein expression of SOX2 are associated with smoking status and histology, and is favorable for prognosis in NSCLC (Li et al., 2016). SOX2 expression is correlated with the expression of proliferation and apoptosis-related proteins and is associated with clinicopathological parameters of worse outcome in primary head and neck squamous cell carcinomas (Schröck et al., 2014). SOX2 can predict prognosis for head and neck squamous cell carcinoma (Chung et al., 2018), and its expression also can be associated with an advanced tumor stage in adenoid cystic carcinoma of the head and neck (Thierauf et al., 2018). SOX2 has also been proved to have anti-proliferative, anti-metastatic, and pro-apoptotic effects. SOX2 is associated with pathological stage and clinical outcome in gastric cancer. SOX2 is the independent prognostic marker for gastric cancer (Wang et al., 2015). The level of SOX2 expression is valuable to predict distant metastasis or the prognosis of nasopharyngeal carcinoma (Wang et al., 2012).

The prognostic value of SOX2 in TNBC is not well-documented. Therefore, it is important to explore the functions and roles of SOX2 in TNBC. Here, we explore the expression, functions and prognostic roles of SOX2 in TNBC and confirm the association of SOX2 in TNBC.

## Materials and Methods

### Cell Lines and Culture

Human normal mammary epithelial cell lines (MCF-10A and 184A1) and breast cancer cell lines (MCF-7, BT474, T47D, MDA-MB-468, BT-20, MDA-MB-435, BT549, and MDA-MB-231) were obtained from the American Type Culture Collection (Manassas, VA, United States) and passaged in our laboratory for less than 6 months after thawing frozen aliquots. All the cell lines were authenticated by short-tandem repeat DNA profiling and all found to be free of mycoplasma infection before use. All cells were maintained according to the supplier’s instructions.

### Clinical Samples

Fresh tissue samples from 20 TNBC tissues (TNBC) and their corresponding paired normal adjacent tissues (Normal), 20 non-triple-negative breast cancer tissues (NTNBC) and their adjacent normal mammal tissues (Control) were cut during surgery and immediately stored in RNAlater (Ambion). The age range of 20 TNBC patients is between 29 and 67, with an average age of (50.7 ± 7.61). The age range of 20 NTNBC patients is between 31 and 64 years, with an average age of (51.3 ± 8.22). There was no significant difference in the basic data between the two groups of patients. These tissue samples were subjected to quantitative real-time polymerase chain reaction (qRT-PCR) analysis. Another 237 TNBC tissues were collected during surgery to be formalin-fixed and embedded in paraffin and then subjected to immunohistochemistry (IHC). All clinical samples were collected between 2006 and 2012 at the Sun Yat-sen University Cancer Center (SYSUCC). The age range of 237 TNBC patients is between 27 and 63, with an average age of (50.93 ± 9.05). This study was approved by the Ethics Committee of SYSUCC Health Authority. The collection and use of tissues followed procedures that are in accordance with the ethical standards formulated in the Declaration of Helsinki. Informed consents were obtained from all patients included in the study.

### qRT-PCR Analysis

The total RNA from all cell lines and tissues were extracted with TRIzol reagent (Invitrogen, Carlsbad, CA, United States). Reverse transcription and qRT-PCR reactions were performed with qSYBR-green-containing PCR kit (Qiagen, United States). The threshold cycle value (CT, the fractional cycle number at which the fluorescence of each sample passes the fixed threshold) of SOX2 was normalized against the CT value of internal control β-actin. The fold change was determined as 2^-ΔΔCt^. The primers for qRT-PCR detection were synthesized by Invitrogen. SOX2 forward, 5′-TAATTAGAATTCATGTA CAACATGATGGAGACG-3′, reverse, 5′-TAATTAGGTACCT CACATGTGTGAGAGGGGCAGTGTGC-3′. The detection was performed with Bio-Rad IQTM5 Multicolour Real-Time PCR Detection System (United States).

### IHC Analysis and Scoring System

After deparaffinization and rehydration, the slides were treated with 90% methanol/3% H_2_O_2_ solution for 10 min at room temperature to block endogenous peroxidase. Then, the slides were soaked in sodium citrate buffer (10 mM Sodium citrate, 0.05% Tween 20, pH 6.0) under 96°C for 5 min for antigen retrieval. After blocking by BSA, antibody against SOX2 (Santa Cruz, CA, United States) was used. We added antibody to the slides for overnight storage at 4°C and then incubated the slides at room temperature with biotinylated secondary antibody for 10 min, and finally HRP-Streptavidin for 10 min. After DAB staining, the results were graded for intensity. The intensities of SOX2 staining were scored between 0 and 4 according to the standards of 0–1 (no staining), 1–2 (weak staining), 2–3 (medium staining), and 3–4 (strong staining). The percentages of SOX2 positive cells in 3 representative high-power fields of individual samples were analyzed. Scores of intensity multiplied by the percentages of positive cells equalled to the final scores of SOX2 expression. The maximum score was 4 and the minimum score was 0. Individual samples were evaluated by three pathologists in a blinded manner, and those expression scores greater or equal to 2 were defined as high expression, less than 2 was defined as low expression.

### Establishment of Stably Transfected Cell Lines

Recombinant shRNA lentiviruses containing sh-SOX2 and sh-control were purchased from FulenGen (Guangzhou, Guangdong, China), Four shRNA sequences were respectively used to knock down SOX2 in MDA-MB-231 and BT549. The relative SOX2 mRNA expression after transfection was respectively showed in **Supplementary Figure S2, S3**. MDA-MB-231 and BT549 cells were respectively infected with sh-SOX2 or sh-control in 24-well plates with the medium changed every 24 h. Cells were selected with minimum lethal concentration of 5 mg/L puromycin (Invivogen, San Diego, CA, United States) for 10 days till drug-resistant cells were obtained. Then these stably transfected cells were used in the following *in vivo* or *in vitro* experiments.

### Cell Proliferation Assay

MDA-MB-231 and BT549 cells respectively infected with sh-SOX2 or sh-control were plated in 6-well plates at a desired cell concentration. The number of cells was counted at 24, 48, 72, and 96 h after incubation by Coulter Counter (Beckman Coulter, Fullerton, CA, United States) in triplicate.

### Cell Invasion Assay

MDA-MB-231 and BT549 cells infected with sh-SOX2 or sh-control were seeded in the upper chamber with Matrigel in the insert of a 24-well culture plate (BD Biosciences, Bedford, MA, United States) with serum-free medium. Then the lower chamber was added with DMEM medium with 15% fetal bovine serum as a chemoattractant. Then the invasive cells adhering to the lower membrane of the inserts were stained with Crystal Violet after 48 h of incubation. Al last the invasive cells were counted and imaged with OLYMPUS IX71 Inverted Microscope (Olympus, Japan, Image-Pro Plus7.0 imaging system).

### Apoptosis Assay

5 × 10^5^ of MDA-MB-231 and BT549 cells infected with sh-SOX2 or sh-control were collected and washed twice with ice-cold PBS. The cells were treated with Alexa Fluor^®^488 annexin V/Dead Cell Apoptosis Kit (Invitrogen, Carlsbad, CA, United States) for Flow Cytometry analysis according to manufacturer’s instructions. The negative control for the double staining was untreated cells. The apoptosis ability of MDA-MB-231 and BT-549 were immediately detected by a FACSCalibur instrument (Becton Dickinson, San Diego, CA, United States).

### Mouse Xenograft Model

5 × 10^6^ MDA-MB-231 cells infected with sh-SOX2 or sh-control were inoculated subcutaneously into the dorsal flanks of nude mice (6 mice in each group). The mice were sacrificed after 28 days, then necropsies were performed, and the tumors were weighed. Then IHC was used to detect the expression of SOX2 in tumor tissues of nude mice. In order to explore the effect of sh-SOX2 on tumor metastasis, 5 × 10^5^ MDA-MB-231 cells infected with sh-SOX2 or sh-control were injected into the tail vein of nude mice (5 mice in each group). The mice were sacrificed 28 days later, and then necropsies were performed. The number of micrometastases in lung tissues per HE-stained section of every individual mice were analyzed by morphological observation with microscope. All procedures for handling animals were performed according with the institutional guidelines and all possible steps were taken to avoid animal suffering at all stages during the experiment.

### Statistical Analysis

All statistical analyses were performed by SPSS16.0 software. *t*-Test and χ^2^ test were used to compare the data between groups. Kaplan–Meier method and Log-rank test were used to plot Overall survival (OS) and disease free survival (DFS) curves. Survival was counted from the day of surgery. The differences were considered statistically significant when *p* < 0.05.

## Results

### SOX2 Was Overexpressed and Correlated With Poor Clinical Outcomes in TNBC

**FIGURE 1:**
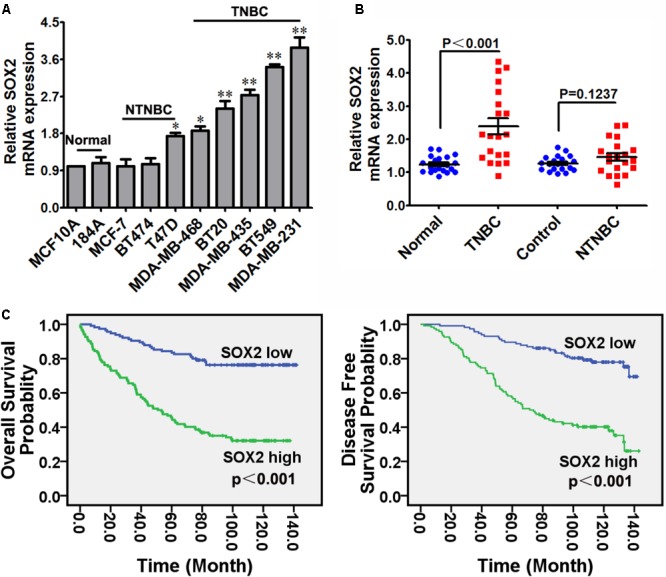
SOX2 overexpressed in TNBC cells and tissues, and correlated with poor clinical outcomes in TNBC. **(A)** Expression of SOX2 in 4 TNBC cell lines, 4 NTNBC cell lines and 2 mammary normal cell lines detected by qRT-PCR. β-actin was used as an internal control (^∗^*p* < 0.05, ^∗∗^*p* < 0.01). **(B)** Expression of SOX2 in 20 TNBC tissues and their corresponding paired normal adjacent tissues (Normal), 20 NTNBC tissues and their corresponding paired normal adjacent tissues (Control). **(C)** OS and DFS curves for 237 cases of TNBC patients with high or low level of SOX2 expression.

The expression of SOX2 in eight breast cancer cell lines (five TNBC cell lines, three NTNBC cell lines) and two mammary normal cell lines were detected by qRT-PCR. The results showed that SOX2 was strongly expressed in all 8 breast cancer cell lines, particularly in TNBC cell lines (**Figure 1A** and **Supplementary Figure S1**): The SOX2 expression levels for some of the TNBC, NTNBC or normal mammary cell lines. The expression of SOX2 in 20 TNBC tissues and their matched adjacent normal tissues (Normal), 20 NTNBC tissues and their matched adjacent normal tissues (Control) were also detected by qRT-PCR. The results showed that approximately 95% (19/20) of the tissues in TNBC group showed notable increase in SOX2 expression compared with the average expression in Normal group (*p* < 0.001, **Figure 1B**). However, there was no significant increase of SOX2 expression in NTNBC tissues compared with the Control group (*p* = 0.1237, **Figure 1B**). These data indicated that SOX2 was mainly overexpressed in TNBC.

In order to determine the significance of SOX2 in clinical prognosis of TNBC, we performed IHC to evaluate SOX2 expression in 237 TNBC tissues. The 237 cases of tissues were divided into low or high groups based on the level of SOX2 expression. The presentative IHC images of three staining degrees (weak-medium-strong) of SOX2 expression under a microscope were showed in **Supplementary Figure S4** (400X). Then OS and DFS curves of these TNBC patients were performed by Kaplan–Meier survival analysis. The results indicated that patients with high SOX2 expression exhibited shorter time of OS (*p* < 0.001) and DFS (*p* < 0.001) than patients with low SOX2 expression (**Figure 1C**). These data indicated that overexpression of SOX2 was significantly associated with poor clinical outcomes of TNBC.

### SOX2 Inhibition Reduced Cell Proliferation and Invasion, and Promoted Cell Apoptosis in TNBC

**FIGURE 2:**
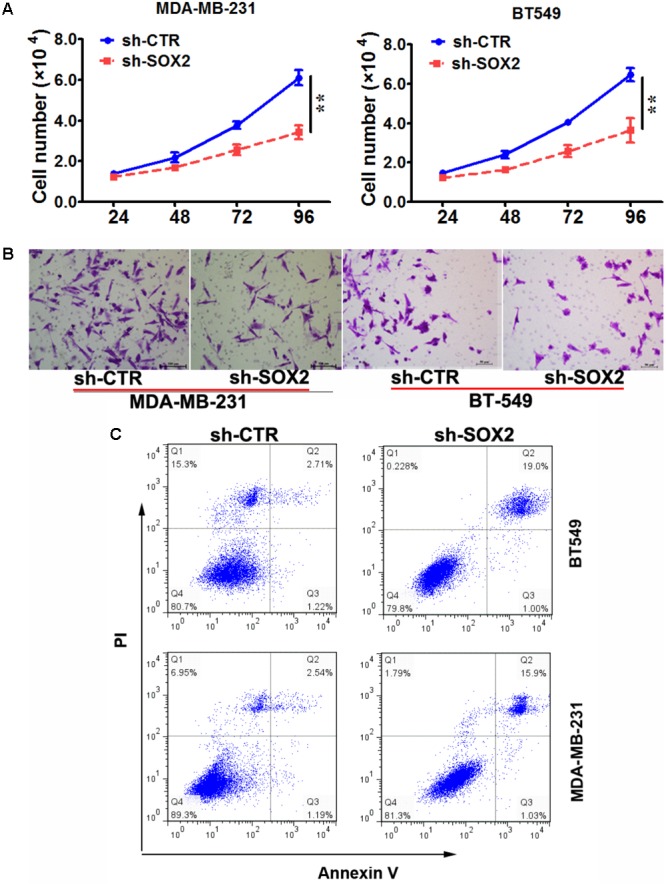
SOX2 inhibition reduced cell proliferation and invasion, and promoted cell apoptosis in TNBC. **(A)** MDA-MB-231 and BT-549 cells were infected with sh-SOX2 or sh-control lentivirus. Cell number was counted at 24, 48, 72, 96 h after infection (^∗∗^*p* < 0.01). **(B)** The invasion ability of MDA-MB-231 and BT-549 cells infected with sh-SOX2 or sh-control lentivirus detected by transwell assays. **(C)** The apoptosis ability of MDA-MB-231 and BT-549 cells infected with sh-SOX2 or sh-control lentivirus detected by apoptosis assays.

The above results indicated an inverse correlation between the SOX2 expression and the clinical outcomes of TNBC. We hypothesized that inhibition of SOX2 expression may improve the malignant status of TNBC. Therefore, we explore the role of SOX2 in proliferation, invasion, and apoptosis in TNBC cell lines. Two TNBC cell lines (MDA-MB-231 and BT549) were respectively infected with sh-SOX2 or sh-control lentivirus. Then the proliferation ability of these two cell lines was detected by MTT assay. We found that the cell numbers in sh-SOX2 infected group (sh-SOX2) were significantly reduced compared with sh-control infected group (sh-CTR) (**Figure 2A**). Meanwhile, the invasion ability of cells was detected by Transwell assay. As expected, the invasion ability of sh-SOX2 group was significantly decreased compared with sh-CTR group (**Figure 2B**). Furthermore, the apoptosis ability of cells was tested by Apoptosis assay. Consistent with our hypothesis, the number of apoptosis cells of sh-SOX2 group was obviously more than those in sh-CTR group (**Figure 2C**).

### SOX2 Inhibition Reduced Tumorigenesis and Metastasis in Xenograft Model

**FIGURE 3:**
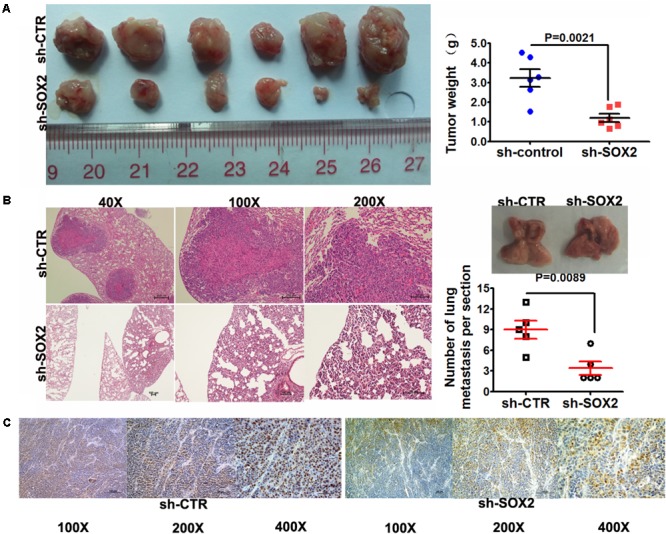
SOX2 inhibition reduced tumorigenesis and metastasis in xenograft model. **(A)** Tumor xenografts in mice. MDA-MB-231 cells infected with sh-SOX2 or sh-control were subcutaneously injected into nude mice (6 in each group). 28 days later, the mice were sacrificed and dissected, and the tumors were weighed. **(B)** Tumor metastasis in mouse xenograft models. MDA-MB-231 cells infected with sh-SOX2 or sh-control lentivirus were injected into the tail vein of nude mice (5 in each group). 28 days later, the mice were sacrificed and micrometastases in the lung per HE-stained section from individual mice were calculated. **(C)** Expression levels of SOX2 in the mouse xenograft model. The expression level of SOX2 in the sh-SOX2 group was significantly lower than that in the sh-CTR group.

To further evaluate the role of SOX2 in tumor formation and growth *in vivo*, we adopted xenograft model of human TNBC cells in nude mice. MDA-MB-231 cells infected with sh-SOX2 or sh-control lentivirus were injected subcutaneously into nude mice (6 mice in each group). All mice were sacrificed to harvest the xenograft tumors after 28 days. The results showed that the mean volume and weight of tumors generated from sh-SOX2 group were both significantly lower than sh-control group (**Figure 3A**). Then the effect of SOX2 on tumor metastasis *in vivo* was performed by metastatic model of human TNBC cells in nude mice. MDA-MB-231 cells infected with sh-SOX2 or sh-control lentivirus were transplanted into the nude mice via tail vein injection. The mice were anesthetized after 28 days and all lungs were dissected. Hematoxylin and eosin (HE) staining was performed to evaluate the tissue morphology. The result showed that the number of macroscopic lung metastases and the SOX2 expression levels observed in sh-SOX2 group was significantly lower than sh-CTR group (**Figures 3B,C**). These results showed that SOX2 inhibition reduced tumorigenesis and metastasis in TNBC.

### Increased SOX2 Levels Were Correlated With Tumor Status, TNM Stage, and Lymph Nodes Infiltration

We explored the potential clinicopathological implications when the expression of SOX2 altered. The expression level of SOX2 was inversely correlated with tumor status, TNM stage and lymph nodes infiltration (three *p*-values were less than 0.001), but not correlated with the patients’ age, menopause, tumor size, and histological grade (four *p*-values were more than 0.05) among these 237 TNBC patients (**Table 1**). These results revealed that SOX2 may play a vital role in the occurrence and progression of TNBC.

**Table 1:** Clinicopathological variables and SOX2 expression in 237 TNBC patients.

Variables	Total (*n* = 237)	SOX2 low (*n* = 115)		SOX2 high (*n* = 122)		*p*-Value
		No.	%	No.	%	
**Age (years)**						0.578
< 50	142	71	50.0	71	50.0	
> = 50	95	44	46.3	51	53.7	
**Menopause**						0.884
Yes	98	47	48.0	51	52.0	
No	139	68	48.9	71	51.1	
**Tumor size (cm)**						0.117
= < 2	75	42	56.0	33	44.0	
>2	162	73	45.1	89	54.9	
**Tumor status (T)**						<0.001^∗∗^
T_1-2_	202	109	54.0	93	46.0	
T_3-4_	35	6	17.1	29	82.9	
**TNM stage**						<0.001^∗∗^
I–II	167	99	59.3	68	40.7	
III–IV	70	16	22.9	54	77.1	
**LN infiltrated**						<0.001^∗∗^
Yes	114	24	21.1	90	78.9	
No	123	91	74.0	32	26.0	
**Histological grade**						0.218
G1	3	2	66.7	1	33.3	
G2	115	60	52.2	55	47.8	
G3	119	53	44.5	66	55.5	

*^∗∗^Statistically significant (*p* < 0.01); % means percentage within the row. LNs, lymph nodes; G1, well-differentiated; G2, moderately differentiated; G3, poorly differentiated.*

## Discussion

Breast cancer is the most common malignancy and the second most common cause of cancer death among female malignant neoplasms (Huang et al., 2016). TNBC is a highly aggressive subcategory of breast cancer. The lack of well-defined molecular targets leads to no effective and readily available targeted therapies for treatment of TNBC (Mayer et al., 2014). In addition, tumor relapse, metastasis and drug resistance also render standard chemotherapy ineffective in the treatment of TNBC (Schröck et al., 2014). Search for specific molecular biomarkers for the treatment of TNBC has become an important area of research for both basic scientists and clinicians.

The acquisition of metastatic phenotypes of various cancers has been linked to the alterations of SOX2 expression. SOX2 is frequently up regulated in cancers and related with worse outcomes. It is reported that SOX2 promotes metastasis through the induction of the epithelial–mesenchymal transition (EMT) (Li et al., 2015). High expression of SOX2 has been correlated with tumor progression of oral squamous cell carcinoma (Fu et al., 2016), hepatocellular carcinoma (Zhao et al., 2015), colorectal cancer (Zheng et al., 2017), glioblastoma (Dong et al., 2017) and others. In this study, we found that SOX2 was up-regulated in both TNBC cell lines and clinical tissues by qRT-PCR. We also found that high expression of SOX2 was associated with shorter OS and DFS. We evaluated the immunohistochemical expression of SOX2 in 237 cases of TNBC tissues and assessed their prognostic significance. We found that SOX2 expression showed a significant association with tumor status, TNM stage and lymph nodes infiltration. All these results are consistent with the existing reports that high expression of SOX2 is associated with poor OS in intrahepatic cholangiocarcinomas (Gu and Jang, 2014). SOX2 was reported to be a prognostic indicator of tongue squamous cell carcinoma (Huang et al., 2014). These results suggest that SOX2 could be a prognostic biomarker for TNBC as well.

Furthermore, we found that SOX2 inhibition reduced cell proliferation and invasion, induced cell apoptosis *in vitro*. SOX2 inhibition also suppressed tumorigenesis and metastasis *in vivo*. These results indicate that SOX2 can act as a tumor promoter in TNBC. Inhibition the expression of SOX2 by recombinant shRNA lentiviruses containing sh-SOX2 can improve the alleviate malignancy of TNBC. All these results are consistent with the existing studies that silencing SOX2 expression by RNA interference can inhibit the proliferation, invasion and metastasis, and induces apoptosis in human laryngeal cancer (Yang N. et al., 2015). Our results demonstrate that SOX2 has a tremendous potential to be a therapeutic target against TNBC.

## Conclusion

In summary, inhibiting the expression of SOX2 can reduce the malignancy state of TNBC including proliferation, invasion, and metastasis. Our findings reveal the biological functions of SOX2 in TNBC. SOX2 is a valuable biomarker for TNBC prognosis and could be a potential therapeutic target of TNBC.

## Author Contributions

HT and CS designed and carried out the experiments. CS interpreted the data and wrote the manuscript. PL, JW, BC, XH, XP, and LL collected the human samples and clinical data.

## Conflict of Interest Statement

The authors declare that the research was conducted in the absence of any commercial or financial relationships that could be construed as a potential conflict of interest.
