# Supplementary material for: SOX2 Promotes Cell Proliferation and Metastasis in Triple Negative Breast Cancer
Source: Front Pharmacol. 2018 Aug 21;9:942. doi: 10.3389/fphar.2018.00942 (PMC6110877; doi:10.3389/fphar.2018.00942)
Supplement: Supplementary file 1 [file Presentation_1.PDF]

Supplement Figure legends

Supplement Figure S1: The SOX2 expression levels for some of the TNBC, NTNBC or normal mammary cell lines.

Supplement Figure S2: Four shRNA sequences were used to knock down SOX2 in MDA-MB-231. The relative SOX2 mRNA expression after transfection. All of the data are shown as the means ± s.e.m. * $P < 0.05$.

Supplement Figure S3: Four shRNA sequences were used to knock down SOX2 in BT-549. The relative SOX2 mRNA expression after transfection. All of the data are shown as the means ± s.e.m. * $P < 0.05$.

Supplement Figure S4: The presentative Immunohistochemical images of three staining degrees (weak-medium-strong) of SOX2 expression under a microscope were showed in Supplement S4 (400X).
