# Supplementary figures and images for: SOX2 Promotes Cell Proliferation and Metastasis in Triple Negative Breast Cancer
Source: Front Pharmacol. 2018 Aug 21;9:942. doi: 10.3389/fphar.2018.00942 (PMC6110877; doi:10.3389/fphar.2018.00942)

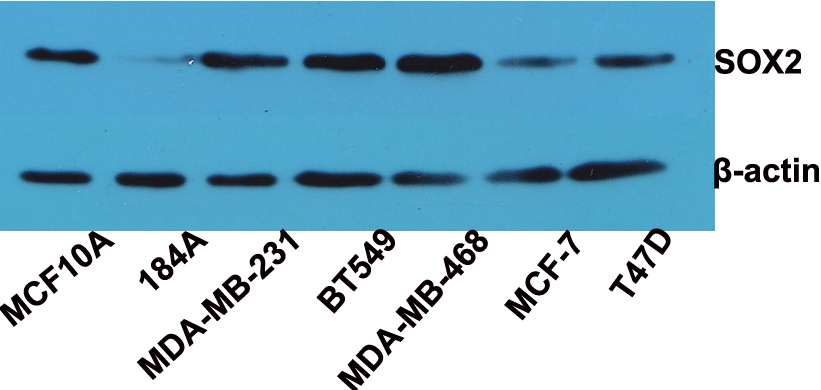

Supplement: Supplementary file 2 [file Image_1.TIF]

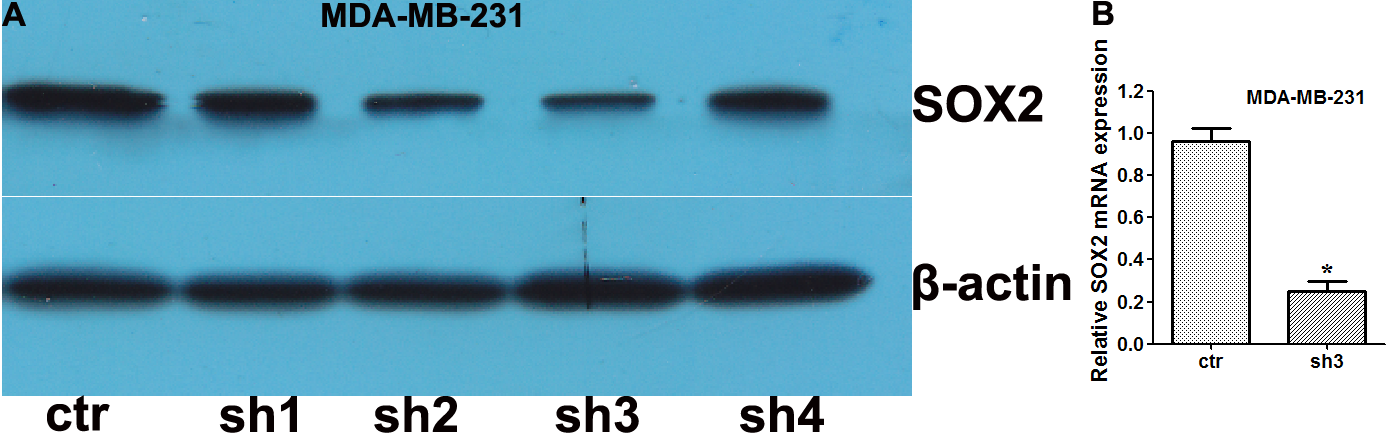

Supplement: Supplementary file 3 [file Image_2.TIF]

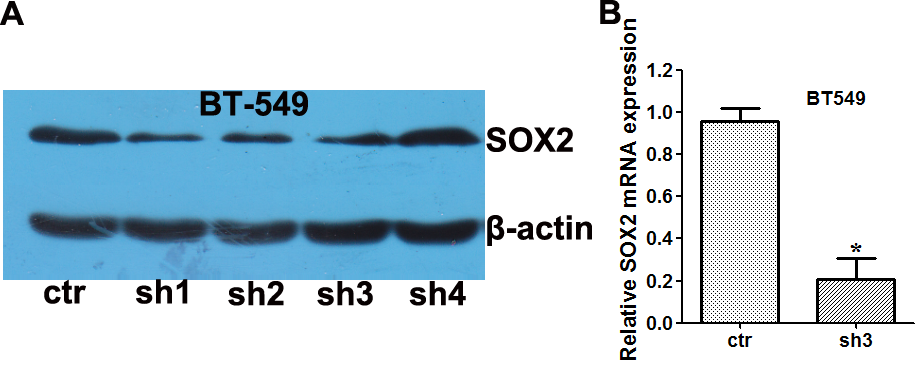

Supplement: Supplementary file 4 [file Image_3.TIF]

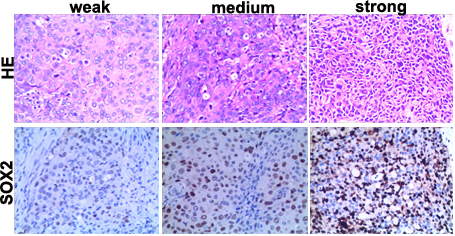

Supplement: Supplementary file 5 [file Image_4.TIF]
